# Supplementary material for: The impact of social distancing on COVID19 spread: State of Georgia case study
Source: PLoS One. 2020 Oct 12;15(10):e0239798. doi: 10.1371/journal.pone.0239798 (PMC7549801; doi:10.1371/journal.pone.0239798)
Supplement: S1 Appendix — (DOCX) [file pone.0239798.s001.docx]

**The impact of social distancing on COVID19 spread: State of Georgia case study supporting information**

**S1 Appendix. Data sources, model description and model inputs**

**Data sources**

Multiple sources of data were used throughout this study, including household type [1], household size [2], children status [2, 3], workflow [4], and population demographics [2]. The household type represents the percentage of households with a specific number of people that are designated as family. The children status is the percentage of households with at least a certain number of children. The workflow is the number of people who live in one census tract and work in another census tract. Our workflow data also includes people who live outside of Georgia, but work in some census tract in Georgia. For the population demographics, we divided the population into five age categories: 0 year olds to 4 year olds, 5 year olds to 9 year olds, 10 year olds to 19 year olds, 20 year olds to 64 year olds, and 65+ year olds.

To ensure that certain groups interacted with certain other groups more, we imposed different probabilities based on the age of the person for the following parameters in the model: probability of hospitalization and probability of death. We were unable to find the proportion of patients of the specific age categories we desired for the following parameters in the literature: proportion of patients that develop symptoms after being exposed, proportion of patients that are asymptomatic after being exposed, and transmission rate.

County-level confirmed COVID19 cases and deaths were collected from The New York Times [2], based on reports from state and local health agencies. County-level hospitalizations were acquired from the Georgia Department of Health [2, 5].

**Agent-based infection spread model**

We adapted a simulation-based disease spread model assuming heterogeneous population mixing to predict the spread pattern of the disease geographically over a period of one year based on existing agent-based simulation models [6-9]. The underlying model was a Susceptible-Exposed-Infected-Recovered (SEIR) model that tracks the disease status of an individual as the disease spreads through a census-tract level contact network by interactions in households, workplaces, schools, and communities. Each population member was assumed to be an agent in the disease spread but with different interactions in the household, workplace and in the community, with different rates of transmission and with different rates for severe outcomes such as hospitalizations and deaths varying with the age group. The model assumed one million agents, that is, one agent corresponding to approximately 10 people in the population in Georgia.

The main assumptions of the model used were (1) every individual is in exactly one of the following states at any given time: susceptible (S), exposed (E), pre-symptomatic (IP), asymptomatic (IA), symptomatic (IS), hospitalized (H), recovered (R), or dead (D) and (2) the entire population has three levels of mixing: (i) community (day and night), (ii) peer groups (day), and (iii) household (night). Other assumptions are made as well when it comes to the various parameters set and how the virus works. Anyone who follows shelter-in-place order does not have any peer-to-peer interaction until the shelter-in-place order is lifted. For those over the age of 64, workplace peer-to-peer interactions end. Everyone between the ages of 20 and 64, inclusive, act the same and have the same likelihood to be infected or spread the virus.

The input model parameters were: probability of developing symptoms stratified by age; probability of hospitalization stratified by age; probability of death given the patient was hospitalized stratified by age; $R_{0}$, reproductive number; $\beta$, transmission rate; the average length of time before a patient becomes pre-symptomatic; the standard deviation for the length of time before a patient becomes pre-symptomatic, the average length of time a patient is hospitalized, average length of time a patient is pre-symptomatic, the probability of isolating a person who has already been hospitalized, the probability of being asymptomatic; $\theta$, the proportion of transmission that occurs at the pre-symptomatic or asymptomatic stage; $\omega,$ the proportion of infections generated by those who are asymptomatic; and the average length of time a patient has symptoms. S1 Table provides the input model parameters along with the references citing these parameters.

**S1 Table. Model parameters.**

| **Parameters** | **Estimates** | **References** |
| --- | --- | --- |
| Probability of Symptomatic | 0.50-0.82 | [10-14] |
| Probability of Hospitalization | 0.016 for age 0-19,  0.18 for age 20-64,  0.30 for age 65+ | [15] |
| Probability of Death | 0 for age 0-19,  0.0515 for age 20-64,  0.3512 for age 65+ | [15] |
| $R_{0}$ | 2.4 | [16-18] |
|  | 2.3 | [19] |
| $\beta$ | 1.12 | [17] |
| Exposed Duration | Weibull with mean 4.6 days | [20, 21] |
| Pre-symptomatic Duration | 0.5 days | [21] |
| Hospitalized Duration | Exponential with mean 10.4 days | [21, 22] |
| Symptomatic Duration | Exponential with mean 2.9 days | [23] |
| Symptomatic-Asymptomatic Duration Ratio | 1.5 | \| [21] \| \| --- \| |
| $\theta$ | 0.48 | \| [24] \| \| --- \| |
| $\omega$ | 0.24 | [24] |
| Percentage of hospitalizations that require ICU | 0% for age 0-19,  20.53% for age 20-64,  28.11% for age 65+ | [15] |
| Percentage of ICU patients that require ventilation | 64% | [5] |
| ICU Duration (days) | 8 | [25] |
| Ventilation Duration (days) | 6 | [26] |

Descriptions and references for the model input parameters.

The path every person takes in the infection spread process starts at being susceptible (unless they start in the exposed phase initially). Anyone who is susceptible has a probability of being exposed based on age group (0-4, 5-9, 10-19, 20-65, 65+) and with whom a person interacts (peer group in school, peer group at work, etc.). Once someone is exposed, he/she will eventually become infectious, called the pre-symptomatic phase. There is some latency between when they are exposed and when they are pre-symptomatic. That duration is based on a Weibull distribution with a specified mean value (in number of days), called *Exposed Duration* in S1 Table. The pre-symptomatic phase lasts 12 hours (0.5 days), the end of which marks when the person is symptomatic or asymptomatic, determined by a fixed probability, *Probability of Symptomatic* in S1 Table. The people who are asymptomatic will always recover but will remain infectious for a variable amount of time (in days) based on an exponential distribution with specified mean, called *Symptomatic Duration* in S1 Table. Any person who is symptomatic can either recover or go to the hospital with an age-dependent probability defined by *Probability of Hospitalization* in S1 Table. Any person who goes to the hospital can, again, either recover, or can die with an age-dependent probability named *Probability of Death* in S1 Table. At each point where there are two options, there is an associated probability given in S1 Table*, Probability of symptomatic, Probability of Hospitalization,* and *Probability of death.*

The *Probability of Symptomatic* in S1 Table is the probability that a pre-symptomatic infected patient will start showing symptoms. The *Probability of Hospitalization* in S1 Table is the probability that a symptomatic patient becomes hospitalized. The *Probability of Death* in S1 Table is the probability that a person who is hospitalized dies. The $R_{0}$in S1 Table is the *reproductive number*, which measures the transmission potential of the virus (i.e., the expected number of secondary infections caused by a typical infection). The $\beta$ in S1 Table parameter represents the transmission rate. The *Exposed Duration* in S1 Table is the length of time (in days) between when a person was exposed to COVID19 from another infectious person and when this person becomes infectious, that is, when a person enters the pre-symptomatic phase. Note that a person becomes infectious when they enter the pre-symptomatic phase. The *Pre-symptomatic Duration* in S1 Table is the amount of time (in days) before an infected patient enters either the symptomatic or asymptomatic phase. Note that a patient will either become symptomatic or asymptomatic after the same amount of time (in days) through the pre-symptomatic phase. Also note that a person who goes from pre-symptomatic to asymptomatic has no fundamental change. It is simply that this person will continue to never develop symptoms. The *Symptomatic Duration* in S1 Table is the amount of time (in days) it takes for a symptomatic person to become either hospitalized or recovered. The *Hospitalized Duration* in S1 Table is the amount of time (in days) a person who has been hospitalized will either become recovered or will die. The *Symptomatic-Asymptomatic Duration Ratio* in S1 Table is the ratio of duration times between symptomatic and asymptomatic, which is used to identify the average asymptomatic duration. The $\theta$ parameter in S1 Table is the proportion of transmission that occurs at the pre-symptomatic or asymptomatic phase. The $\omega$ parameter in S1 Table denotes the proportion of infections generated by individuals who are asymptomatic.

To seed the model, we utilized the confirmed case data for Georgia. Since the case data was stratified down to the county level and our initialization needed data down to the census tract level, we took the numbers provided for the county level from The New York Times and applied the Huntington-Hill method of apportionment (the same method used to decide the number of seats that belong to each U.S. congressman in the House of Representatives) to apportion the number of COVID19 confirmed cases from March 24th to each of the census tracts in the state of Georgia. We used the distribution of the seeds from the confirmed cases to inform the community-level projections.

At the start of a simulation run, an initial infection was introduced randomly to agents from census tracts following the distributions of confirmed cases in Georgia. Any susceptible individual who becomes infected changes their disease status from susceptible to exposed. With pre-defined probabilities, the disease progresses within infected individuals and spreads to previously healthy individuals across the network. Once recovered from the disease, the individual remains in that state as there is no definitive evidence that it is possible to be re-infected with COVID19 after full recovery.

The code used was implemented using C++.

**S1 Fig. Model descriptions.** Agent-base model incorporates the natural history of the disease for each individual agent, by age group, and the interactions at the household, peer group, and community, across different geographic areas.

**Capacity need estimation: description of model inputs**

To calculate the daily number of hospital beds needed, we take the needs from the previous day after removing patients that have been discharged and add the daily new hospitalizations by age group. The number of patients who are discharged is determined by taking a fraction of the hospitalized population, based on the mean value of the *Hospitalization Duration*. The *Hospitalized Duration* is defined as the average length of stay of a COVID19 patient in the hospital. Calculations for ICU bed needs are found by taking the ICU patients from the previous day after removing individuals that have been discharged from the ICU and adding a percentage of the daily new hospitalizations by age group, where percentages are determined by the age-dependent *Percentage of hospitalizations that require ICU* from S1 Table*.* The *Percentage of hospitalizations that require ICU* is the percentage of COVID19 hospital patients that have been transferred to the ICU because of the severity of their illness. This percentage has been considered to be age-dependent and the estimations for each age group are given in S1 Table*.* The number of ICU patients that are discharged is a fraction of the current COVID19 ICU population, based on the *ICU Duration* from S1 Table*.* The *ICU Duration* is the average length of stay in the ICU for a COVID19 patient. Lastly, the calculations for ventilator needs are determined by taking the ICU patients from the previous day that are on ventilation after removing the ICU patients who have stopped ventilation and adding a percentage of the daily new ICU patients, based on the *Percentage of ICU patients that require ventilation* from S1 Table*.* The *Percentage of ICU patients that require ventilation* is defined as the percent of COVID19 patients in the ICU whose illness severity requires respiratory support through mechanical ventilation or ECMO services. The fraction of ventilation patients who are removed from ventilation services is calculated from the average length of time on ventilation for a COVID19 patient, referred to as *Ventilation Duration* in S1 Table*.*

**Calibration and validation of the model**

SIP intervention at the state level in Georgia was not implemented on March 16th; however most of the businesses, profit and non-profit organizations, and governmental organizations have enacted various interventions to establish VQ; starting on April 3, 2020 [27], the entire state of Georgia enacted statewide directives for SIP until April 30, 2020; new guidance was issued by the Governor of Georgia on April 20, 2020 that gyms, bowling alleys, tattoo parlors, barbers, hair and nail salons, and massage therapists may reopen for business on April 24, 2020, and theaters and dine-in restaurants may reopen for business on April 27, 2020 [28].

Due to high variations in preliminary COVID19 estimates, we calibrated our model and adjusted disease parameters according to confirmed COVID19 infections, hospitalizations and deaths in the state of Georgia. Percentage of symptomatic cases was adjusted based on the literature and confirmed cases. Probability of hospitalization was calculated and adjusted based on CDC report [15] and [5] to capture the age specific hospitalization probabilities and better mimic the current condition in the state of Georgia, to that end we multiplied probabilities provided in [11] with 1/3. In *Figure B1*, we present the cumulative number of deaths confirmed by the state of Georgia in comparison to our projections and the confirmed COVID19 cases in Georgia multiplied by 8 to account for under-testing and existence of asymptomatic cases. Georgia results are shifted one week earlier to account for the infection incubation period in comparison to our daily infection estimates.

One way to assess the validity of the model was to examine the how our model performed on urban versus rural counties. For example, DeKalb County, an urban county, experiences the earliest peak day on June 29^th^ under Scenario 1, but under Scenario 4 or 7, the peak shifted to July 9^th^-18^th^. Increasing VQ compliance to mid or high would push the peak day to July 7^th^ and August 3^rd^, respectively. On the other hand, Lanier County, a rural county, has its earliest peak on July 6^th^ under Scenario 1, but under Scenario 4 or 7, the peak shifts to July 15^th^-29^th^. However, increasing VQ compliance to mid or high would push the peak day to July 15^th^ and August 11^th^.

For instance, peak infection percentage in Fulton County increases from 0.81% to 0.82% from Scenario 1 to 4 and decreases from 0.82% to 0.79% from Scenario 4 to 7. Similar fluctuations can be observed in other urban and rural counties. The rural county of Webster has a peak percentage decrease from 0.82% to 0.57% from Scenario 1 to 4 and increase from 0.57% to 0.74% from Scenario 4 to 7. Increasing VQ compliance from low to mid and from mid to high provides approximately a 10% and 26-37% decrease, respectively, in peak infection percentage.

County-level risk factors were calculated and are visualized in S2 Fig*.*

**S2 Fig. County-level risk factor.** County-level risk factor (left) and its multiplier (right) derived by applying the principal component analysis on several factors known to impact a higher risk of complications and severe outcomes for COVID19 infections, including prevalence of asthma, diabetes, obesity, smoking, cardiovascular disease and chronic conditions in general

**S2 Appendix. Supplemental figures, tables and results**

This supplemental material provides additional figures and tables complementing the results presented in the main manuscript.

**S3 Fig. Cumulative number of COVID19 deaths and infections.** Cumulative number of COVID19 deaths (left plot) and infections (right plot) of Scenarios 1, 2, 3 with respect to confirmed numbers of Georgia. On left, the actual COVID19 deaths in Georgia is plotted whereas on right, the confirmed COVID19 cases in Georgia multiplied by 8 to account for under-testing and existence of asymptomatic cases and moved one week earlier to account for incubation period.

**S4 Fig. State level outcomes: IAR, CAR, ISOR, IFR, HB, ICUB, V across all scenarios.** State Level Outcomes: IAR (first row left plot), CAR (first row right plot), ISOR (second row left plot), IFR (second row right plot), hospital bed capacity (third row left plot), ICUB (third row right plot), V (fourth row center plot) across all scenarios (including the baseline scenarios).

**S5 Fig. State level outcomes: NIC for low, mid, and high levels of VQ after 4, 5, and 6 weeks of SIP.** State level Outcomes: Daily new COVID19 infections when Low VQ is combined with 4 week (Scenario 1), 5 week (Scenario 4), 6 week (Scenario 7) SIP (top left plot), Mid VQ is combined with 4 week (Scenario 2), 5 week (Scenario 5), 6 week (Scenario 8) SIP (top right plot), High VQ is combined with 4 week (Scenario 3), 5 week (Scenario 6), 6 week (Scenario 9) SIP (bottom center plot).

**S6 Fig. Number of new cases per 100,000 people.** Four maps of Georgia at the county level recording the number of new cases per 100,000 people for April 23,2020 (using the actual number of cases1), May 15, 2020 (simulated data from our model), June 15, 2020 (simulated data), and July 15, 2020 (simulated data).

**S7 Fig. Hospital regions.** Georgia map of the 14 coordinating hospital regions.

**S2 Table. Peak day in each county.**

| **County Name** | **NI** | **SC** | **1** | **2** | **3** | **4** | **5** | **6** | **7** | **8** | **9** |
| --- | --- | --- | --- | --- | --- | --- | --- | --- | --- | --- | --- |
| Appling | 30-Apr | 10-May | 30-Jun | 28-Jul | 12-Aug | 27-Jul | 12-Aug | 14-Aug | 4-Aug | 12-Aug | 15-Aug |
| Atkinson | 27-Apr | 29-Apr | 2-Jul | 16-Jul | 10-Aug | 19-Jul | 2-Aug | 6-Aug | 25-Jul | 29-Jul | 13-Aug |
| Bacon | 21-Apr | 9-May | 16-Jul | 21-Jul | 4-Aug | 19-Jul | 2-Aug | 15-Aug | 29-Jul | 9-Aug | 15-Aug |
| Baker | 1-May | 28-Apr | 20-Jul | 17-Jul | 15-Aug | 26-Jul | 14-Jul | 13-Aug | 29-Jul | 4-Aug | 27-Jul |
| Baldwin | 30-Apr | 6-May | 14-Jul | 19-Jul | 31-Jul | 19-Jul | 25-Jul | 15-Aug | 27-Jul | 9-Aug | 15-Aug |
| Banks | 22-Apr | 24-Apr | 1-Jul | 7-Jul | 10-Aug | 6-Jul | 11-Jul | 12-Aug | 14-Jul | 24-Jul | 10-Aug |
| Barrow | 17-Apr | 21-Apr | 28-Jun | 10-Jul | 14-Aug | 8-Jul | 18-Jul | 9-Aug | 18-Jul | 28-Jul | 15-Aug |
| Bartow | 17-Apr | 25-Apr | 30-Jun | 6-Jul | 5-Aug | 9-Jul | 12-Jul | 11-Aug | 16-Jul | 26-Jul | 15-Aug |
| Ben Hill | 28-Apr | 1-May | 26-Jul | 22-Jul | 13-Aug | 6-Aug | 12-Aug | 15-Aug | 9-Aug | 9-Aug | 15-Aug |
| Berrien | 25-Apr | 29-Apr | 16-Jul | 18-Jul | 10-Aug | 13-Jul | 27-Jul | 14-Aug | 29-Jul | 1-Aug | 13-Aug |
| Bibb | 26-Apr | 1-May | 5-Jul | 17-Jul | 8-Aug | 21-Jul | 24-Jul | 10-Aug | 24-Jul | 3-Aug | 14-Aug |
| Bleckley | 18-Apr | 27-Apr | 10-Jul | 22-Jul | 15-Aug | 28-Jul | 3-Aug | 12-Aug | 24-Jul | 11-Aug | 13-Aug |
| Brantley | 24-Apr | 26-Apr | 30-Jun | 25-Jul | 7-Aug | 16-Jul | 21-Jul | 13-Aug | 21-Jul | 12-Aug | 15-Aug |
| Brooks | 21-Apr | 26-Apr | 22-Jul | 17-Jul | 12-Aug | 21-Jul | 4-Aug | 9-Aug | 27-Jul | 14-Aug | 15-Aug |
| Bryan | 19-Apr | 27-Apr | 5-Jul | 24-Jul | 11-Aug | 17-Jul | 15-Jul | 15-Aug | 15-Jul | 1-Aug | 13-Aug |
| Bulloch | 17-Apr | 30-Apr | 1-Jul | 20-Jul | 7-Aug | 21-Jul | 28-Jul | 15-Aug | 22-Jul | 5-Aug | 15-Aug |
| Burke | 25-Apr | 29-Apr | 16-Jul | 24-Jul | 13-Aug | 21-Jul | 22-Jul | 9-Aug | 31-Jul | 6-Aug | 14-Aug |
| Butts | 16-Apr | 27-Apr | 26-Jun | 7-Jul | 3-Aug | 9-Jul | 11-Jul | 13-Aug | 12-Jul | 4-Aug | 10-Aug |
| Calhoun | 28-Apr | 27-Apr | 23-Jul | 18-Jul | 14-Aug | 13-Jul | 24-Jul | 9-Aug | 9-Aug | 8-Aug | 15-Aug |
| Camden | 25-Apr | 30-Apr | 20-Jul | 2-Aug | 14-Aug | 9-Jul | 26-Jul | 15-Aug | 27-Jul | 8-Aug | 14-Aug |
| Candler | 26-Apr | 3-May | 7-Jul | 26-Jul | 8-Aug | 12-Jul | 1-Aug | 13-Aug | 22-Jul | 8-Aug | 13-Aug |
| Carroll | 16-Apr | 26-Apr | 29-Jun | 12-Jul | 4-Aug | 13-Jul | 15-Jul | 11-Aug | 19-Jul | 1-Aug | 14-Aug |
| Catoosa | 23-Apr | 26-Apr | 7-Jul | 15-Jul | 8-Aug | 15-Jul | 23-Jul | 12-Aug | 25-Jul | 31-Jul | 15-Aug |
| Charlton | 5-May | 27-Apr | 28-Jul | 30-Jul | 14-Aug | 20-Jul | 5-Aug | 15-Aug | 12-Aug | 5-Aug | 15-Aug |
| Chatham | 23-Apr | 28-Apr | 5-Jul | 14-Jul | 7-Aug | 20-Jul | 20-Jul | 13-Aug | 22-Jul | 4-Aug | 15-Aug |
| Chattahoochee | 28-Apr | 11-May | 12-Jul | 15-Jul | 6-Aug | 29-Jul | 31-Jul | 10-Aug | 3-Aug | 10-Aug | 13-Aug |
| Chattooga | 22-Apr | 24-Apr | 8-Jul | 23-Jul | 12-Aug | 13-Jul | 24-Jul | 15-Aug | 24-Jul | 30-Jul | 15-Aug |
| Cherokee | 16-Apr | 22-Apr | 1-Jul | 7-Jul | 3-Aug | 8-Jul | 15-Jul | 15-Aug | 20-Jul | 24-Jul | 14-Aug |
| Clarke | 20-Apr | 24-Apr | 3-Jul | 10-Jul | 10-Aug | 7-Jul | 26-Jul | 15-Aug | 24-Jul | 31-Jul | 15-Aug |
| Clay | 5-May | 7-May | 14-Jul | 29-Jul | 8-Aug | 27-Jul | 9-Aug | 15-Aug | 11-Aug | 8-Aug | 2-Aug |
| Clayton | 15-Apr | 25-Apr | 30-Jun | 3-Jul | 1-Aug | 7-Jul | 18-Jul | 13-Aug | 15-Jul | 27-Jul | 14-Aug |
| Clinch | 1-May | 11-May | 25-Jul | 5-Aug | 31-Jul | 27-Jul | 8-Aug | 15-Aug | 4-Aug | 13-Aug | 11-Aug |
| Cobb | 16-Apr | 23-Apr | 2-Jul | 7-Jul | 30-Jul | 4-Jul | 16-Jul | 14-Aug | 16-Jul | 25-Jul | 12-Aug |
| Coffee | 22-Apr | 1-May | 15-Jul | 2-Aug | 8-Aug | 25-Jul | 4-Aug | 12-Aug | 29-Jul | 3-Aug | 14-Aug |
| Colquitt | 29-Apr | 29-Apr | 10-Jul | 17-Jul | 10-Aug | 28-Jul | 26-Jul | 15-Aug | 28-Jul | 8-Aug | 14-Aug |
| Columbia | 20-Apr | 26-Apr | 7-Jul | 15-Jul | 13-Aug | 13-Jul | 24-Jul | 15-Aug | 24-Jul | 5-Aug | 15-Aug |
| Cook | 27-Apr | 3-May | 12-Jul | 25-Jul | 12-Aug | 16-Jul | 27-Jul | 12-Aug | 22-Jul | 9-Aug | 13-Aug |
| Coweta | 16-Apr | 24-Apr | 30-Jun | 6-Jul | 3-Aug | 13-Jul | 13-Jul | 15-Aug | 20-Jul | 28-Jul | 15-Aug |
| Crawford | 25-Apr | 2-May | 5-Jul | 15-Jul | 15-Aug | 13-Jul | 28-Jul | 13-Aug | 26-Jul | 13-Aug | 14-Aug |
| Crisp | 24-Apr | 6-May | 13-Jul | 28-Jul | 8-Aug | 18-Jul | 3-Aug | 11-Aug | 28-Jul | 12-Aug | 12-Aug |
| Dade | 25-Apr | 11-May | 18-Jul | 28-Jul | 15-Aug | 5-Aug | 29-Jul | 15-Aug | 29-Jul | 9-Aug | 15-Aug |
| Dawson | 18-Apr | 24-Apr | 28-Jun | 1-Jul | 30-Jul | 13-Jul | 16-Jul | 4-Aug | 18-Jul | 28-Jul | 15-Aug |
| Decatur | 1-May | 6-May | 12-Jul | 28-Jul | 13-Aug | 28-Jul | 26-Jul | 10-Aug | 13-Aug | 8-Aug | 15-Aug |
| DeKalb | 17-Apr | 21-Apr | 29-Jun | 7-Jul | 3-Aug | 9-Jul | 17-Jul | 10-Aug | 18-Jul | 28-Jul | 15-Aug |
| Dodge | 18-Apr | 30-Apr | 10-Jul | 25-Jul | 14-Aug | 20-Jul | 30-Jul | 4-Aug | 29-Jul | 5-Aug | 12-Aug |
| Dooly | 26-Apr | 28-Apr | 8-Jul | 8-Jul | 2-Aug | 23-Jul | 31-Jul | 4-Aug | 14-Jul | 9-Aug | 13-Aug |
| Dougherty | 24-Apr | 25-Apr | 9-Jul | 20-Jul | 13-Aug | 20-Jul | 31-Jul | 15-Aug | 29-Jul | 5-Aug | 15-Aug |
| Douglas | 15-Apr | 23-Apr | 30-Jun | 7-Jul | 28-Jul | 7-Jul | 20-Jul | 11-Aug | 16-Jul | 29-Jul | 14-Aug |
| Early | 3-May | 3-May | 24-Jul | 29-Jul | 15-Aug | 31-Jul | 9-Aug | 15-Aug | 14-Aug | 1-Aug | 15-Aug |
| Echols | 23-Apr | 8-May | 15-Jul | 25-Jul | 12-Aug | 15-Jul | 5-Aug | 12-Aug | 1-Aug | 30-Jul | 13-Aug |
| Effingham | 19-Apr | 26-Apr | 4-Jul | 19-Jul | 11-Aug | 12-Jul | 27-Jul | 15-Aug | 20-Jul | 1-Aug | 12-Aug |
| Elbert | 22-Apr | 1-May | 8-Jul | 19-Jul | 15-Aug | 8-Jul | 30-Jul | 15-Aug | 26-Jul | 7-Aug | 13-Aug |
| Emanuel | 25-Apr | 24-Apr | 13-Jul | 25-Jul | 8-Aug | 2-Aug | 29-Jul | 12-Aug | 5-Aug | 5-Aug | 11-Aug |
| Evans | 23-Apr | 25-Apr | 14-Jul | 2-Jul | 4-Aug | 17-Jul | 19-Jul | 29-Jul | 26-Jul | 13-Aug | 13-Aug |
| Fannin | 29-Apr | 6-May | 7-Jul | 27-Jul | 8-Aug | 27-Jul | 17-Jul | 15-Aug | 23-Jul | 9-Aug | 15-Aug |
| Fayette | 17-Apr | 25-Apr | 1-Jul | 8-Jul | 4-Aug | 11-Jul | 15-Jul | 12-Aug | 15-Jul | 23-Jul | 15-Aug |
| Floyd | 20-Apr | 29-Apr | 2-Jul | 8-Jul | 14-Aug | 13-Jul | 26-Jul | 14-Aug | 25-Jul | 3-Aug | 15-Aug |
| Forsyth | 19-Apr | 22-Apr | 27-Jun | 10-Jul | 31-Jul | 12-Jul | 15-Jul | 8-Aug | 16-Jul | 28-Jul | 13-Aug |
| Franklin | 23-Apr | 26-Apr | 26-Jun | 14-Jul | 12-Aug | 13-Jul | 22-Jul | 12-Aug | 22-Jul | 27-Jul | 13-Aug |
| Fulton | 17-Apr | 25-Apr | 29-Jun | 8-Jul | 3-Aug | 9-Jul | 17-Jul | 11-Aug | 16-Jul | 25-Jul | 15-Aug |
| Gilmer | 22-Apr | 28-Apr | 6-Jul | 11-Jul | 13-Aug | 9-Jul | 15-Jul | 15-Aug | 21-Jul | 5-Aug | 14-Aug |
| Glascock | 29-Apr | 5-May | 7-Jul | 25-Jul | 8-Aug | 26-Jul | 2-Aug | 12-Aug | 19-Jul | 10-Aug | 11-Aug |
| Glynn | 26-Apr | 26-Apr | 14-Jul | 23-Jul | 10-Aug | 11-Jul | 5-Aug | 15-Aug | 24-Jul | 4-Aug | 14-Aug |
| Gordon | 19-Apr | 25-Apr | 30-Jun | 5-Jul | 15-Aug | 16-Jul | 17-Jul | 11-Aug | 15-Jul | 25-Jul | 14-Aug |
| Grady | 30-Apr | 8-May | 19-Jul | 23-Jul | 8-Aug | 27-Jul | 1-Aug | 15-Aug | 4-Aug | 2-Aug | 14-Aug |
| Greene | 28-Apr | 26-Apr | 18-Jul | 12-Jul | 15-Aug | 18-Jul | 27-Jul | 11-Aug | 2-Aug | 7-Aug | 14-Aug |
| Gwinnett | 15-Apr | 23-Apr | 25-Jun | 5-Jul | 2-Aug | 6-Jul | 14-Jul | 13-Aug | 18-Jul | 26-Jul | 15-Aug |
| Habersham | 22-Apr | 23-Apr | 28-Jun | 3-Jul | 5-Aug | 11-Jul | 25-Jul | 7-Aug | 22-Jul | 28-Jul | 15-Aug |
| Hall | 16-Apr | 21-Apr | 30-Jun | 6-Jul | 5-Aug | 7-Jul | 11-Jul | 13-Aug | 15-Jul | 24-Jul | 15-Aug |
| Hancock | 2-May | 2-May | 23-Jul | 21-Jul | 11-Aug | 24-Jul | 4-Aug | 11-Aug | 22-Jul | 2-Aug | 14-Aug |
| Haralson | 21-Apr | 28-Apr | 27-Jun | 10-Jul | 3-Aug | 12-Jul | 19-Jul | 10-Aug | 22-Jul | 30-Jul | 13-Aug |
| Harris | 19-Apr | 4-May | 6-Jul | 17-Jul | 9-Aug | 21-Jul | 1-Aug | 14-Aug | 25-Jul | 1-Aug | 15-Aug |
| Hart | 26-Apr | 28-Apr | 9-Jul | 15-Jul | 15-Aug | 14-Jul | 27-Jul | 14-Aug | 29-Jul | 6-Aug | 13-Aug |
| Heard | 23-Apr | 26-Apr | 1-Jul | 5-Jul | 12-Aug | 10-Jul | 3-Aug | 15-Aug | 12-Jul | 26-Jul | 15-Aug |
| Henry | 15-Apr | 22-Apr | 27-Jun | 7-Jul | 1-Aug | 4-Jul | 16-Jul | 13-Aug | 15-Jul | 26-Jul | 14-Aug |
| Houston | 23-Apr | 27-Apr | 2-Jul | 8-Jul | 5-Aug | 15-Jul | 19-Jul | 9-Aug | 18-Jul | 3-Aug | 15-Aug |
| Irwin | 25-Apr | 1-May | 10-Jul | 27-Jul | 13-Aug | 23-Jul | 24-Jul | 6-Aug | 30-Jul | 12-Aug | 13-Aug |
| Jackson | 17-Apr | 20-Apr | 30-Jun | 8-Jul | 7-Aug | 4-Jul | 18-Jul | 12-Aug | 20-Jul | 21-Jul | 14-Aug |
| Jasper | 18-Apr | 23-Apr | 29-Jun | 14-Jul | 11-Aug | 8-Jul | 17-Jul | 8-Aug | 21-Jul | 5-Aug | 7-Aug |
| Jeff Davis | 30-Apr | 7-May | 7-Jul | 26-Jul | 13-Aug | 29-Jul | 12-Aug | 4-Aug | 24-Jul | 13-Aug | 12-Aug |
| Jefferson | 28-Apr | 2-May | 20-Jul | 30-Jul | 15-Aug | 23-Jul | 2-Aug | 15-Aug | 22-Jul | 6-Aug | 15-Aug |
| Jenkins | 27-Apr | 2-May | 12-Jul | 9-Jul | 10-Aug | 22-Jul | 14-Jul | 1-Aug | 24-Jul | 3-Aug | 14-Aug |
| Johnson | 26-Apr | 1-May | 13-Jul | 28-Jul | 13-Aug | 26-Jul | 26-Jul | 15-Aug | 5-Aug | 14-Aug | 15-Aug |
| Jones | 26-Apr | 30-Apr | 8-Jul | 13-Jul | 12-Aug | 21-Jul | 31-Jul | 12-Aug | 29-Jul | 29-Jul | 15-Aug |
| Lamar | 20-Apr | 25-Apr | 9-Jul | 10-Jul | 11-Aug | 8-Jul | 22-Jul | 14-Aug | 27-Jul | 1-Aug | 14-Aug |
| Lanier | 25-Apr | 4-May | 6-Jul | 15-Jul | 11-Aug | 15-Jul | 25-Jul | 13-Aug | 29-Jul | 8-Aug | 15-Aug |
| Laurens | 21-Apr | 29-Apr | 18-Jul | 25-Jul | 15-Aug | 13-Jul | 1-Aug | 15-Aug | 28-Jul | 10-Aug | 14-Aug |
| Lee | 26-Apr | 20-Apr | 14-Jul | 15-Jul | 10-Aug | 22-Jul | 7-Aug | 15-Aug | 28-Jul | 4-Aug | 14-Aug |
| Liberty | 20-Apr | 26-Apr | 23-Jun | 11-Jul | 25-Jul | 17-Jul | 20-Jul | 11-Aug | 16-Jul | 4-Aug | 15-Aug |
| Lincoln | 28-Apr | 6-May | 9-Jul | 19-Jul | 7-Aug | 16-Jul | 28-Jul | 15-Aug | 2-Aug | 10-Aug | 14-Aug |
| Long | 24-Apr | 27-Apr | 19-Jun | 14-Jul | 4-Aug | 13-Jul | 18-Jul | 15-Aug | 20-Jul | 27-Jul | 12-Aug |
| Lowndes | 22-Apr | 26-Apr | 10-Jul | 22-Jul | 9-Aug | 20-Jul | 9-Aug | 13-Aug | 28-Jul | 7-Aug | 13-Aug |
| Lumpkin | 23-Apr | 26-Apr | 3-Jul | 11-Jul | 7-Aug | 7-Jul | 18-Jul | 12-Aug | 25-Jul | 24-Jul | 13-Aug |
| McDuffie | 25-Apr | 1-May | 9-Jul | 26-Jul | 1-Aug | 21-Jul | 27-Jul | 13-Aug | 26-Jul | 4-Aug | 13-Aug |
| McIntosh | 26-Apr | 28-Apr | 11-Jul | 20-Jul | 3-Aug | 17-Jul | 28-Jul | 15-Aug | 19-Jul | 5-Aug | 14-Aug |
| Macon | 1-May | 3-May | 4-Jul | 24-Jul | 8-Aug | 20-Jul | 18-Jul | 2-Aug | 1-Aug | 24-Jul | 15-Aug |
| Madison | 21-Apr | 27-Apr | 4-Jul | 13-Jul | 7-Aug | 15-Jul | 24-Jul | 13-Aug | 28-Jul | 4-Aug | 15-Aug |
| Marion | 29-Apr | 26-Apr | 17-Jul | 21-Jul | 10-Aug | 7-Aug | 1-Aug | 14-Aug | 10-Aug | 3-Aug | 14-Aug |
| Meriwether | 24-Apr | 27-Apr | 23-Jun | 17-Jul | 15-Aug | 12-Jul | 24-Jul | 15-Aug | 21-Jul | 5-Aug | 13-Aug |
| Miller | 1-May | 8-May | 19-Jul | 31-Jul | 14-Aug | 28-Jul | 10-Aug | 30-Jul | 8-Aug | 15-Aug | 15-Aug |
| Mitchell | 28-Apr | 27-Apr | 13-Jul | 23-Jul | 12-Aug | 26-Jul | 27-Jul | 13-Aug | 2-Aug | 12-Aug | 14-Aug |
| Monroe | 22-Apr | 2-May | 28-Jun | 19-Jul | 8-Aug | 14-Jul | 27-Jul | 10-Aug | 21-Jul | 2-Aug | 13-Aug |
| Montgomery | 21-Apr | 12-May | 8-Jul | 2-Aug | 11-Aug | 23-Jul | 30-Jul | 13-Aug | 7-Aug | 13-Aug | 14-Aug |
| Morgan | 20-Apr | 26-Apr | 24-Jun | 19-Jul | 31-Jul | 18-Jul | 14-Jul | 12-Aug | 24-Jul | 28-Jul | 13-Aug |
| Murray | 17-Apr | 22-Apr | 30-Jun | 7-Jul | 3-Aug | 9-Jul | 21-Jul | 15-Aug | 16-Jul | 30-Jul | 14-Aug |
| Muscogee | 25-Apr | 3-May | 10-Jul | 23-Jul | 15-Aug | 25-Jul | 2-Aug | 15-Aug | 28-Jul | 9-Aug | 14-Aug |
| Newton | 16-Apr | 26-Apr | 28-Jun | 5-Jul | 27-Jul | 11-Jul | 14-Jul | 9-Aug | 17-Jul | 22-Jul | 15-Aug |
| Oconee | 16-Apr | 22-Apr | 30-Jun | 18-Jul | 11-Aug | 9-Jul | 30-Jul | 12-Aug | 21-Jul | 24-Jul | 13-Aug |
| Oglethorpe | 17-Apr | 19-Apr | 5-Jul | 7-Jul | 15-Aug | 12-Jul | 20-Jul | 13-Aug | 26-Jul | 18-Jul | 14-Aug |
| Paulding | 16-Apr | 22-Apr | 27-Jun | 5-Jul | 30-Jul | 5-Jul | 13-Jul | 8-Aug | 17-Jul | 29-Jul | 12-Aug |
| Peach | 24-Apr | 1-May | 2-Jul | 10-Jul | 6-Aug | 1-Aug | 22-Jul | 15-Aug | 26-Jul | 5-Aug | 14-Aug |
| Pickens | 21-Apr | 19-Apr | 27-Jun | 11-Jul | 6-Aug | 11-Jul | 19-Jul | 10-Aug | 17-Jul | 6-Aug | 13-Aug |
| Pierce | 26-Apr | 3-May | 15-Jul | 30-Jul | 2-Aug | 24-Jul | 25-Jul | 12-Aug | 2-Aug | 8-Aug | 14-Aug |
| Pike | 17-Apr | 22-Apr | 7-Jul | 18-Jul | 6-Aug | 19-Jul | 18-Jul | 14-Aug | 20-Jul | 28-Jul | 13-Aug |
| Polk | 19-Apr | 30-Apr | 5-Jul | 12-Jul | 7-Aug | 10-Jul | 27-Jul | 12-Aug | 11-Jul | 2-Aug | 15-Aug |
| Pulaski | 24-Apr | 30-Apr | 15-Jul | 19-Jul | 4-Aug | 11-Jul | 4-Aug | 3-Aug | 26-Jul | 11-Aug | 15-Aug |
| Putnam | 23-Apr | 2-May | 6-Jul | 17-Jul | 2-Aug | 12-Jul | 26-Jul | 7-Aug | 30-Jul | 25-Jul | 15-Aug |
| Quitman | 7-May | 7-May | 8-Aug | 21-Jul | 11-Aug | 28-Jul | 27-Jul | 2-Aug | 9-Aug | 31-Jul | 15-Aug |
| Rabun | 29-Apr | 10-May | 13-Jul | 19-Jul | 13-Aug | 19-Jul | 12-Aug | 15-Aug | 31-Jul | 27-Jul | 15-Aug |
| Randolph | 15-May | 18-May | 24-Jul | 5-Aug | 9-Aug | 10-Aug | 15-Aug | 14-Aug | 13-Aug | 1-Aug | 14-Aug |
| Richmond | 22-Apr | 30-Apr | 11-Jul | 18-Jul | 11-Aug | 10-Jul | 27-Jul | 14-Aug | 25-Jul | 5-Aug | 15-Aug |
| Rockdale | 16-Apr | 25-Apr | 29-Jun | 7-Jul | 4-Aug | 5-Jul | 19-Jul | 12-Aug | 17-Jul | 29-Jul | 15-Aug |
| Schley | 24-Apr | 6-May | 13-Jul | 22-Jul | 13-Aug | 26-Jul | 30-Jul | 4-Aug | 1-Aug | 6-Aug | 14-Aug |
| Screven | 20-Apr | 1-May | 14-Jul | 27-Jul | 12-Aug | 20-Jul | 19-Jul | 11-Aug | 21-Jul | 12-Aug | 15-Aug |
| Seminole | 4-May | 3-May | 24-Jul | 7-Aug | 14-Aug | 23-Jul | 4-Aug | 31-Jul | 12-Aug | 12-Aug | 15-Aug |
| Spalding | 19-Apr | 25-Apr | 25-Jun | 5-Jul | 6-Aug | 7-Jul | 24-Jul | 13-Aug | 14-Jul | 29-Jul | 15-Aug |
| Stephens | 27-Apr | 28-Apr | 6-Jul | 11-Jul | 11-Aug | 17-Jul | 25-Jul | 15-Aug | 24-Jul | 4-Aug | 8-Aug |
| Stewart | 2-May | 9-May | 3-Jul | 20-Jul | 13-Aug | 15-Jul | 8-Aug | 15-Aug | 25-Jul | 14-Aug | 13-Aug |
| Sumter | 25-Apr | 30-Apr | 17-Jul | 18-Jul | 14-Aug | 20-Jul | 31-Jul | 14-Aug | 2-Aug | 15-Aug | 11-Aug |
| Talbot | 23-Apr | 1-May | 9-Jul | 19-Jul | 14-Aug | 22-Jul | 9-Aug | 15-Aug | 31-Jul | 3-Aug | 7-Aug |
| Taliaferro | 25-Apr | 25-Apr | 22-Jul | 19-Jul | 7-Aug | 19-Jul | 10-Aug | 15-Aug | 26-Jul | 1-Aug | 15-Aug |
| Tattnall | 27-Apr | 3-May | 12-Jul | 14-Jul | 9-Aug | 19-Jul | 23-Jul | 15-Aug | 20-Jul | 8-Aug | 14-Aug |
| Taylor | 26-Apr | 4-May | 9-Jul | 9-Jul | 15-Aug | 2-Aug | 2-Aug | 9-Aug | 22-Jul | 6-Aug | 14-Aug |
| Telfair | 27-Apr | 2-May | 13-Jul | 22-Jul | 11-Aug | 19-Jul | 31-Jul | 10-Aug | 27-Jul | 8-Aug | 14-Aug |
| Terrell | 26-Apr | 28-Apr | 15-Jul | 15-Jul | 15-Aug | 31-Jul | 7-Aug | 9-Aug | 11-Aug | 5-Aug | 15-Aug |
| Thomas | 24-Apr | 4-May | 12-Jul | 30-Jul | 15-Aug | 31-Jul | 8-Aug | 13-Aug | 11-Aug | 2-Aug | 14-Aug |
| Tift | 22-Apr | 30-Apr | 2-Jul | 18-Jul | 6-Aug | 15-Jul | 19-Jul | 15-Aug | 27-Jul | 2-Aug | 15-Aug |
| Toombs | 24-Apr | 4-May | 21-Jul | 20-Jul | 6-Aug | 28-Jul | 9-Aug | 14-Aug | 17-Jul | 15-Aug | 15-Aug |
| Towns | 3-May | 20-May | 15-Jul | 20-Jul | 5-Aug | 4-Aug | 4-Aug | 14-Aug | 29-Jul | 14-Aug | 12-Aug |
| Treutlen | 27-Apr | 30-Apr | 10-Jul | 21-Jul | 11-Aug | 24-Jul | 30-Jul | 10-Aug | 18-Jul | 6-Aug | 13-Aug |
| Troup | 21-Apr | 3-May | 6-Jul | 14-Jul | 14-Aug | 20-Jul | 28-Jul | 9-Aug | 22-Jul | 7-Aug | 15-Aug |
| Turner | 29-Apr | 3-May | 8-Jul | 21-Jul | 8-Aug | 17-Jul | 29-Jul | 8-Aug | 24-Jul | 12-Aug | 4-Aug |
| Twiggs | 29-Apr | 1-May | 5-Jul | 16-Jul | 15-Aug | 18-Jul | 23-Jul | 12-Aug | 17-Jul | 31-Jul | 13-Aug |
| Union | 1-May | 9-May | 23-Jul | 30-Jul | 15-Aug | 19-Jul | 15-Aug | 11-Aug | 3-Aug | 8-Aug | 12-Aug |
| Upson | 24-Apr | 1-May | 28-Jun | 21-Jul | 11-Aug | 20-Jul | 21-Jul | 14-Aug | 21-Jul | 4-Aug | 15-Aug |
| Walker | 25-Apr | 2-May | 10-Jul | 20-Jul | 14-Aug | 22-Jul | 23-Jul | 14-Aug | 23-Jul | 28-Jul | 14-Aug |
| Walton | 18-Apr | 24-Apr | 30-Jun | 4-Jul | 4-Aug | 7-Jul | 19-Jul | 8-Aug | 15-Jul | 28-Jul | 13-Aug |
| Ware | 20-Apr | 5-May | 11-Jul | 22-Jul | 13-Aug | 16-Jul | 22-Jul | 15-Aug | 24-Jul | 12-Aug | 15-Aug |
| Warren | 2-May | 9-May | 6-Jul | 27-Jul | 11-Aug | 29-Jul | 27-Jul | 8-Aug | 14-Jul | 8-Aug | 9-Aug |
| Washington | 26-Apr | 5-May | 21-Jul | 6-Aug | 6-Aug | 26-Jul | 28-Jul | 15-Aug | 30-Jul | 10-Aug | 15-Aug |
| Wayne | 22-Apr | 3-May | 7-Jul | 7-Jul | 14-Aug | 17-Jul | 25-Jul | 13-Aug | 22-Jul | 8-Aug | 10-Aug |
| Webster | 3-May | 11-May | 29-Jul | 3-Aug | 13-Aug | 3-Jul | 10-Aug | 1-Aug | 1-Aug | 26-Jul | 14-Aug |
| Wheeler | 22-Apr | 3-May | 18-Jul | 20-Jul | 11-Aug | 14-Jul | 6-Aug | 5-Aug | 21-Jul | 11-Aug | 13-Aug |
| White | 20-Apr | 27-Apr | 1-Jul | 5-Jul | 6-Aug | 10-Jul | 18-Jul | 6-Aug | 24-Jul | 25-Jul | 14-Aug |
| Whitfield | 20-Apr | 22-Apr | 30-Jun | 14-Jul | 7-Aug | 7-Jul | 16-Jul | 15-Aug | 21-Jul | 27-Jul | 15-Aug |
| Wilcox | 28-Apr | 7-May | 8-Jul | 8-Jul | 14-Aug | 17-Jul | 19-Jul | 10-Aug | 27-Jul | 4-Aug | 15-Aug |
| Wilkes | 30-Apr | 10-May | 7-Jul | 27-Jul | 14-Aug | 2-Aug | 10-Aug | 23-Jul | 1-Aug | 11-Aug | 15-Aug |
| Wilkinson | 25-Apr | 6-May | 5-Jul | 25-Jul | 14-Aug | 24-Jul | 26-Jul | 15-Aug | 25-Jul | 14-Aug | 15-Aug |
| Worth | 24-Apr | 2-May | 10-Jul | 25-Jul | 4-Aug | 24-Jul | 26-Jul | 14-Aug | 28-Jul | 10-Aug | 14-Aug |

Peak Day in each county in the State of Georgia under all scenarios tested with urban counties highlighted in green.

**S3 Table. Peak infection percentage in each county.**

| **County Name** | **NI** | **SC** | **1** | **2** | **3** | **4** | **5** | **6** | **7** | **8** | **9** |
| --- | --- | --- | --- | --- | --- | --- | --- | --- | --- | --- | --- |
| Appling | 1.45 | 0.99 | 0.64 | 0.60 | 0.50 | 0.86 | 0.55 | 0.54 | 0.69 | 0.57 | 0.23 |
| Atkinson | 2.10 | 1.18 | 0.75 | 0.65 | 0.50 | 0.94 | 0.71 | 0.48 | 0.84 | 0.69 | 0.37 |
| Bacon | 1.59 | 1.17 | 0.67 | 0.64 | 0.37 | 0.69 | 0.55 | 0.50 | 0.87 | 0.76 | 0.43 |
| Baker | 1.58 | 1.01 | 0.89 | 0.86 | 0.51 | 0.70 | 0.66 | 0.45 | 0.70 | 0.76 | 0.28 |
| Baldwin | 1.55 | 1.16 | 0.76 | 0.58 | 0.46 | 0.74 | 0.65 | 0.53 | 0.80 | 0.74 | 0.45 |
| Banks | 1.82 | 1.29 | 0.88 | 0.76 | 0.58 | 0.92 | 0.70 | 0.62 | 0.80 | 0.76 | 0.49 |
| Barrow | 2.12 | 1.39 | 0.87 | 0.74 | 0.48 | 0.87 | 0.73 | 0.53 | 0.85 | 0.74 | 0.52 |
| Bartow | 2.00 | 1.34 | 0.89 | 0.69 | 0.50 | 0.83 | 0.72 | 0.50 | 0.81 | 0.78 | 0.51 |
| Ben Hill | 1.37 | 1.09 | 0.64 | 0.71 | 0.43 | 0.77 | 0.52 | 0.38 | 0.70 | 0.59 | 0.28 |
| Berrien | 1.79 | 1.03 | 0.63 | 0.72 | 0.46 | 0.83 | 0.66 | 0.52 | 0.74 | 0.73 | 0.35 |
| Bibb | 1.69 | 1.09 | 0.72 | 0.63 | 0.49 | 0.73 | 0.65 | 0.46 | 0.72 | 0.60 | 0.34 |
| Bleckley | 1.75 | 1.10 | 0.79 | 0.71 | 0.55 | 0.76 | 0.63 | 0.51 | 0.81 | 0.67 | 0.36 |
| Brantley | 1.73 | 1.12 | 0.65 | 0.71 | 0.46 | 0.77 | 0.67 | 0.39 | 0.84 | 0.68 | 0.48 |
| Brooks | 1.58 | 1.07 | 0.67 | 0.68 | 0.46 | 0.76 | 0.62 | 0.50 | 0.71 | 0.64 | 0.32 |
| Bryan | 1.90 | 1.13 | 0.71 | 0.56 | 0.43 | 0.80 | 0.72 | 0.46 | 0.76 | 0.65 | 0.44 |
| Bulloch | 1.73 | 1.25 | 0.80 | 0.61 | 0.48 | 0.75 | 0.76 | 0.47 | 0.78 | 0.69 | 0.43 |
| Burke | 1.85 | 1.19 | 0.74 | 0.68 | 0.46 | 0.80 | 0.69 | 0.50 | 0.78 | 0.64 | 0.39 |
| Butts | 2.01 | 1.49 | 0.95 | 0.81 | 0.54 | 0.83 | 0.87 | 0.53 | 0.83 | 0.75 | 0.44 |
| Calhoun | 1.54 | 0.95 | 0.81 | 0.64 | 0.40 | 0.68 | 0.70 | 0.43 | 0.80 | 0.75 | 0.33 |
| Camden | 1.53 | 1.13 | 0.69 | 0.67 | 0.63 | 0.74 | 0.69 | 0.42 | 0.70 | 0.64 | 0.48 |
| Candler | 1.71 | 1.33 | 0.82 | 0.68 | 0.55 | 0.78 | 0.64 | 0.47 | 0.77 | 0.65 | 0.44 |
| Carroll | 2.03 | 1.34 | 0.86 | 0.72 | 0.53 | 0.80 | 0.72 | 0.52 | 0.74 | 0.72 | 0.43 |
| Catoosa | 1.64 | 1.14 | 0.68 | 0.73 | 0.49 | 0.73 | 0.62 | 0.42 | 0.78 | 0.65 | 0.46 |
| Charlton | 1.65 | 1.08 | 0.85 | 0.61 | 0.48 | 0.69 | 0.74 | 0.36 | 0.77 | 0.62 | 0.39 |
| Chatham | 1.74 | 1.18 | 0.74 | 0.63 | 0.44 | 0.78 | 0.68 | 0.46 | 0.75 | 0.63 | 0.43 |
| Chattahoochee | 1.52 | 1.23 | 0.84 | 0.71 | 0.58 | 0.69 | 0.66 | 0.48 | 0.74 | 0.75 | 0.37 |
| Chattooga | 1.89 | 1.26 | 0.73 | 0.71 | 0.45 | 0.83 | 0.67 | 0.49 | 0.82 | 0.65 | 0.41 |
| Cherokee | 2.04 | 1.36 | 0.88 | 0.74 | 0.51 | 0.87 | 0.72 | 0.50 | 0.83 | 0.73 | 0.47 |
| Clarke | 1.85 | 1.23 | 0.80 | 0.69 | 0.52 | 0.82 | 0.71 | 0.47 | 0.81 | 0.67 | 0.45 |
| Clay | 1.41 | 0.86 | 0.86 | 0.60 | 0.26 | 0.70 | 0.71 | 0.43 | 0.63 | 0.60 | 0.20 |
| Clayton | 2.21 | 1.43 | 0.89 | 0.73 | 0.48 | 0.89 | 0.72 | 0.54 | 0.78 | 0.73 | 0.48 |
| Clinch | 1.36 | 1.12 | 0.77 | 0.76 | 0.37 | 0.71 | 0.43 | 0.36 | 0.71 | 0.68 | 0.21 |
| Cobb | 2.06 | 1.38 | 0.86 | 0.72 | 0.50 | 0.85 | 0.72 | 0.52 | 0.83 | 0.73 | 0.50 |
| Coffee | 1.62 | 1.13 | 0.64 | 0.60 | 0.45 | 0.71 | 0.67 | 0.47 | 0.72 | 0.65 | 0.34 |
| Colquitt | 1.50 | 1.04 | 0.68 | 0.65 | 0.53 | 0.71 | 0.63 | 0.27 | 0.63 | 0.58 | 0.38 |
| Columbia | 1.82 | 1.22 | 0.75 | 0.67 | 0.53 | 0.74 | 0.64 | 0.45 | 0.69 | 0.59 | 0.39 |
| Cook | 1.66 | 1.08 | 0.66 | 0.82 | 0.43 | 0.73 | 0.59 | 0.45 | 0.76 | 0.66 | 0.35 |
| Coweta | 2.08 | 1.39 | 0.89 | 0.73 | 0.48 | 0.84 | 0.72 | 0.54 | 0.83 | 0.73 | 0.46 |
| Crawford | 1.82 | 1.17 | 0.78 | 0.72 | 0.47 | 0.76 | 0.70 | 0.52 | 0.74 | 0.59 | 0.41 |
| Crisp | 1.48 | 1.04 | 0.67 | 0.67 | 0.55 | 0.73 | 0.55 | 0.44 | 0.73 | 0.67 | 0.40 |
| Dade | 1.37 | 1.03 | 0.54 | 0.65 | 0.43 | 0.68 | 0.62 | 0.36 | 0.74 | 0.61 | 0.32 |
| Dawson | 1.94 | 1.33 | 0.81 | 0.71 | 0.48 | 0.83 | 0.78 | 0.47 | 0.92 | 0.70 | 0.46 |
| Decatur | 1.59 | 0.98 | 0.61 | 0.58 | 0.37 | 0.70 | 0.65 | 0.41 | 0.63 | 0.67 | 0.26 |
| DeKalb | 2.01 | 1.33 | 0.85 | 0.71 | 0.48 | 0.84 | 0.70 | 0.50 | 0.81 | 0.70 | 0.49 |
| Dodge | 1.53 | 1.16 | 0.73 | 0.65 | 0.48 | 0.70 | 0.62 | 0.43 | 0.69 | 0.68 | 0.34 |
| Dooly | 1.80 | 1.26 | 0.73 | 0.73 | 0.59 | 0.74 | 0.70 | 0.46 | 0.73 | 0.79 | 0.43 |
| Dougherty | 1.58 | 0.94 | 0.66 | 0.63 | 0.46 | 0.67 | 0.60 | 0.37 | 0.66 | 0.56 | 0.29 |
| Douglas | 2.07 | 1.44 | 0.86 | 0.74 | 0.51 | 0.84 | 0.70 | 0.53 | 0.82 | 0.73 | 0.51 |
| Early | 1.42 | 0.94 | 0.63 | 0.55 | 0.45 | 0.76 | 0.58 | 0.36 | 0.69 | 0.59 | 0.19 |
| Echols | 1.79 | 1.11 | 0.84 | 0.77 | 0.78 | 1.01 | 0.85 | 0.63 | 0.95 | 0.86 | 0.55 |
| Effingham | 1.86 | 1.26 | 0.84 | 0.64 | 0.46 | 0.81 | 0.70 | 0.55 | 0.90 | 0.68 | 0.43 |
| Elbert | 1.57 | 1.05 | 0.81 | 0.67 | 0.48 | 0.71 | 0.61 | 0.44 | 0.66 | 0.55 | 0.29 |
| Emanuel | 1.56 | 0.98 | 0.64 | 0.79 | 0.52 | 0.64 | 0.60 | 0.41 | 0.80 | 0.67 | 0.34 |
| Evans | 1.76 | 1.20 | 0.68 | 0.71 | 0.50 | 0.66 | 0.76 | 0.41 | 0.77 | 0.70 | 0.39 |
| Fannin | 1.31 | 0.92 | 0.61 | 0.50 | 0.35 | 0.75 | 0.49 | 0.40 | 0.57 | 0.59 | 0.22 |
| Fayette | 2.00 | 1.28 | 0.84 | 0.73 | 0.49 | 0.83 | 0.67 | 0.53 | 0.80 | 0.71 | 0.43 |
| Floyd | 1.85 | 1.14 | 0.78 | 0.67 | 0.43 | 0.75 | 0.69 | 0.53 | 0.79 | 0.63 | 0.41 |
| Forsyth | 2.20 | 1.41 | 0.92 | 0.78 | 0.52 | 0.87 | 0.73 | 0.49 | 0.85 | 0.76 | 0.50 |
| Franklin | 1.88 | 1.20 | 0.78 | 0.68 | 0.46 | 0.78 | 0.64 | 0.49 | 0.81 | 0.65 | 0.34 |
| Fulton | 1.98 | 1.31 | 0.81 | 0.68 | 0.47 | 0.82 | 0.69 | 0.49 | 0.79 | 0.69 | 0.46 |
| Gilmer | 1.67 | 1.10 | 0.72 | 0.68 | 0.48 | 0.73 | 0.62 | 0.46 | 0.78 | 0.63 | 0.44 |
| Glascock | 2.19 | 1.15 | 0.81 | 0.73 | 0.58 | 0.73 | 0.80 | 0.33 | 0.81 | 0.66 | 0.35 |
| Glynn | 1.55 | 0.92 | 0.66 | 0.62 | 0.40 | 0.74 | 0.62 | 0.40 | 0.72 | 0.67 | 0.38 |
| Gordon | 1.93 | 1.36 | 0.82 | 0.68 | 0.49 | 0.84 | 0.69 | 0.48 | 0.87 | 0.69 | 0.46 |
| Grady | 1.37 | 1.03 | 0.80 | 0.70 | 0.44 | 0.65 | 0.63 | 0.33 | 0.63 | 0.54 | 0.24 |
| Greene | 1.41 | 1.00 | 0.70 | 0.52 | 0.42 | 0.63 | 0.59 | 0.27 | 0.65 | 0.60 | 0.38 |
| Gwinnett | 2.19 | 1.46 | 0.88 | 0.74 | 0.47 | 0.86 | 0.72 | 0.52 | 0.83 | 0.74 | 0.52 |
| Habersham | 1.83 | 1.21 | 0.78 | 0.67 | 0.55 | 0.89 | 0.69 | 0.47 | 0.71 | 0.67 | 0.45 |
| Hall | 2.11 | 1.40 | 0.85 | 0.76 | 0.51 | 0.87 | 0.70 | 0.52 | 0.78 | 0.72 | 0.48 |
| Hancock | 1.63 | 1.07 | 0.74 | 0.57 | 0.52 | 0.66 | 0.74 | 0.45 | 0.86 | 0.76 | 0.49 |
| Haralson | 1.91 | 1.42 | 0.97 | 0.74 | 0.57 | 0.87 | 0.74 | 0.58 | 0.80 | 0.89 | 0.46 |
| Harris | 1.52 | 1.20 | 0.63 | 0.68 | 0.51 | 0.68 | 0.59 | 0.39 | 0.73 | 0.67 | 0.32 |
| Hart | 1.65 | 1.16 | 0.82 | 0.66 | 0.52 | 0.72 | 0.68 | 0.50 | 0.82 | 0.70 | 0.30 |
| Heard | 2.02 | 1.26 | 0.86 | 0.82 | 0.43 | 0.86 | 0.68 | 0.54 | 0.82 | 0.74 | 0.46 |
| Henry | 2.09 | 1.40 | 0.90 | 0.71 | 0.47 | 0.88 | 0.76 | 0.54 | 0.81 | 0.75 | 0.48 |
| Houston | 1.84 | 1.19 | 0.77 | 0.61 | 0.51 | 0.78 | 0.66 | 0.44 | 0.77 | 0.66 | 0.44 |
| Irwin | 1.67 | 1.03 | 0.57 | 0.63 | 0.44 | 0.72 | 0.53 | 0.45 | 0.80 | 0.59 | 0.21 |
| Jackson | 1.96 | 1.39 | 0.81 | 0.72 | 0.48 | 0.84 | 0.70 | 0.48 | 0.84 | 0.71 | 0.48 |
| Jasper | 2.05 | 1.35 | 0.90 | 0.78 | 0.50 | 0.84 | 0.69 | 0.63 | 0.97 | 0.73 | 0.44 |
| Jeff Davis | 1.55 | 1.06 | 0.73 | 0.67 | 0.60 | 0.71 | 0.65 | 0.47 | 0.70 | 0.71 | 0.27 |
| Jefferson | 1.68 | 1.21 | 0.83 | 0.66 | 0.55 | 0.82 | 0.60 | 0.36 | 0.70 | 0.63 | 0.35 |
| Jenkins | 1.86 | 1.38 | 0.75 | 0.73 | 0.47 | 0.70 | 0.73 | 0.44 | 0.74 | 0.57 | 0.47 |
| Johnson | 1.63 | 1.17 | 0.76 | 0.66 | 0.49 | 0.89 | 0.67 | 0.41 | 0.82 | 0.72 | 0.37 |
| Jones | 1.76 | 1.18 | 0.80 | 0.72 | 0.54 | 0.81 | 0.67 | 0.48 | 0.85 | 0.69 | 0.53 |
| Lamar | 1.89 | 1.22 | 0.77 | 0.74 | 0.51 | 0.86 | 0.71 | 0.57 | 0.72 | 0.66 | 0.39 |
| Lanier | 1.71 | 1.11 | 0.81 | 0.73 | 0.47 | 0.70 | 0.71 | 0.59 | 0.81 | 0.76 | 0.43 |
| Laurens | 1.46 | 1.03 | 0.71 | 0.66 | 0.54 | 0.74 | 0.60 | 0.41 | 0.68 | 0.57 | 0.37 |
| Lee | 1.73 | 1.05 | 0.76 | 0.70 | 0.50 | 0.71 | 0.64 | 0.43 | 0.73 | 0.76 | 0.33 |
| Liberty | 1.90 | 1.25 | 0.79 | 0.72 | 0.53 | 0.86 | 0.82 | 0.50 | 0.79 | 0.75 | 0.56 |
| Lincoln | 1.42 | 0.98 | 0.71 | 0.66 | 0.56 | 0.81 | 0.58 | 0.45 | 0.72 | 0.70 | 0.34 |
| Long | 2.00 | 1.35 | 0.79 | 0.85 | 0.61 | 0.87 | 0.83 | 0.60 | 0.91 | 0.76 | 0.57 |
| Lowndes | 1.63 | 1.07 | 0.63 | 0.67 | 0.48 | 0.72 | 0.62 | 0.53 | 0.68 | 0.76 | 0.40 |
| Lumpkin | 1.94 | 1.39 | 0.87 | 0.68 | 0.50 | 0.88 | 0.72 | 0.61 | 0.79 | 0.62 | 0.48 |
| McDuffie | 1.71 | 1.06 | 0.73 | 0.66 | 0.46 | 0.72 | 0.67 | 0.45 | 0.73 | 0.63 | 0.41 |
| McIntosh | 1.59 | 1.08 | 0.67 | 0.63 | 0.50 | 0.76 | 0.60 | 0.45 | 0.74 | 0.64 | 0.42 |
| Macon | 1.59 | 1.00 | 0.63 | 0.56 | 0.43 | 0.74 | 0.65 | 0.33 | 0.73 | 0.57 | 0.43 |
| Madison | 1.86 | 1.21 | 0.92 | 0.71 | 0.52 | 0.88 | 0.74 | 0.53 | 0.83 | 0.70 | 0.53 |
| Marion | 1.66 | 0.99 | 0.74 | 0.61 | 0.40 | 0.81 | 0.62 | 0.42 | 0.74 | 0.58 | 0.36 |
| Meriwether | 1.69 | 1.19 | 0.75 | 0.71 | 0.38 | 0.79 | 0.65 | 0.42 | 0.80 | 0.69 | 0.43 |
| Miller | 1.48 | 1.02 | 0.62 | 0.61 | 0.38 | 0.69 | 0.72 | 0.27 | 0.67 | 0.86 | 0.21 |
| Mitchell | 1.59 | 1.12 | 0.78 | 0.64 | 0.49 | 0.70 | 0.67 | 0.40 | 0.66 | 0.61 | 0.37 |
| Monroe | 1.90 | 1.24 | 0.81 | 0.77 | 0.54 | 0.76 | 0.70 | 0.51 | 0.73 | 0.67 | 0.41 |
| Montgomery | 1.64 | 1.06 | 0.72 | 0.72 | 0.60 | 0.75 | 0.72 | 0.43 | 0.75 | 0.70 | 0.34 |
| Morgan | 1.84 | 1.21 | 0.80 | 0.70 | 0.51 | 0.87 | 0.65 | 0.42 | 0.81 | 0.73 | 0.57 |
| Murray | 1.88 | 1.33 | 0.85 | 0.80 | 0.43 | 0.83 | 0.69 | 0.48 | 0.90 | 0.73 | 0.52 |
| Muscogee | 1.51 | 1.07 | 0.60 | 0.58 | 0.42 | 0.66 | 0.54 | 0.37 | 0.68 | 0.56 | 0.28 |
| Newton | 2.08 | 1.46 | 0.91 | 0.75 | 0.48 | 0.83 | 0.71 | 0.54 | 0.88 | 0.71 | 0.45 |
| Oconee | 2.05 | 1.20 | 0.83 | 0.64 | 0.56 | 0.89 | 0.73 | 0.50 | 0.83 | 0.72 | 0.47 |
| Oglethorpe | 1.86 | 1.18 | 0.79 | 0.69 | 0.59 | 0.83 | 0.71 | 0.44 | 0.79 | 0.70 | 0.43 |
| Paulding | 2.22 | 1.40 | 0.87 | 0.75 | 0.53 | 0.89 | 0.73 | 0.52 | 0.84 | 0.75 | 0.52 |
| Peach | 1.83 | 1.14 | 0.76 | 0.60 | 0.55 | 0.73 | 0.68 | 0.48 | 0.78 | 0.67 | 0.35 |
| Pickens | 1.77 | 1.19 | 0.83 | 0.72 | 0.50 | 0.86 | 0.73 | 0.44 | 0.75 | 0.67 | 0.46 |
| Pierce | 1.48 | 0.96 | 0.69 | 0.71 | 0.44 | 0.75 | 0.60 | 0.37 | 0.69 | 0.56 | 0.39 |
| Pike | 1.85 | 1.34 | 0.74 | 0.75 | 0.50 | 0.95 | 0.72 | 0.54 | 0.92 | 0.80 | 0.45 |
| Polk | 2.01 | 1.32 | 0.86 | 0.68 | 0.51 | 0.78 | 0.73 | 0.47 | 0.75 | 0.71 | 0.46 |
| Pulaski | 1.72 | 1.28 | 0.72 | 0.68 | 0.55 | 0.77 | 0.61 | 0.47 | 0.78 | 0.85 | 0.43 |
| Putnam | 1.60 | 1.11 | 0.81 | 0.61 | 0.49 | 0.67 | 0.57 | 0.48 | 0.77 | 0.69 | 0.42 |
| Quitman | 1.17 | 0.85 | 0.55 | 0.43 | 0.50 | 0.82 | 0.55 | 0.27 | 0.69 | 0.62 | 0.30 |
| Rabun | 1.27 | 0.93 | 0.64 | 0.56 | 0.41 | 0.59 | 0.55 | 0.42 | 0.60 | 0.51 | 0.22 |
| Randolph | 1.13 | 0.65 | 0.50 | 0.43 | 0.24 | 0.60 | 0.43 | 0.18 | 0.41 | 0.35 | 0.22 |
| Richmond | 1.67 | 1.18 | 0.72 | 0.65 | 0.50 | 0.71 | 0.61 | 0.44 | 0.67 | 0.56 | 0.39 |
| Rockdale | 2.13 | 1.39 | 0.92 | 0.70 | 0.51 | 0.83 | 0.73 | 0.49 | 0.84 | 0.73 | 0.49 |
| Schley | 1.86 | 1.00 | 0.84 | 0.73 | 0.33 | 0.80 | 0.60 | 0.33 | 0.83 | 0.73 | 0.40 |
| Screven | 1.71 | 1.19 | 0.89 | 0.65 | 0.43 | 0.79 | 0.77 | 0.39 | 0.90 | 0.64 | 0.35 |
| Seminole | 1.56 | 0.92 | 0.56 | 0.53 | 0.31 | 0.64 | 0.67 | 0.29 | 0.73 | 0.60 | 0.18 |
| Spalding | 1.93 | 1.32 | 0.84 | 0.77 | 0.48 | 0.85 | 0.73 | 0.54 | 0.82 | 0.70 | 0.43 |
| Stephens | 1.85 | 1.17 | 0.73 | 0.73 | 0.46 | 0.74 | 0.64 | 0.51 | 0.70 | 0.83 | 0.36 |
| Stewart | 1.54 | 1.30 | 0.76 | 0.74 | 0.56 | 0.77 | 0.66 | 0.56 | 0.73 | 0.57 | 0.31 |
| Sumter | 1.65 | 0.94 | 0.66 | 0.64 | 0.43 | 0.77 | 0.65 | 0.39 | 0.65 | 0.67 | 0.31 |
| Talbot | 1.57 | 1.13 | 0.75 | 0.67 | 0.36 | 0.79 | 0.72 | 0.33 | 0.83 | 0.54 | 0.34 |
| Taliaferro | 1.68 | 1.11 | 0.76 | 0.87 | 0.73 | 0.84 | 0.57 | 0.49 | 0.73 | 0.92 | 0.33 |
| Tattnall | 1.86 | 1.17 | 0.76 | 0.77 | 0.59 | 0.81 | 0.80 | 0.51 | 0.74 | 0.80 | 0.45 |
| Taylor | 1.62 | 1.04 | 0.83 | 0.66 | 0.46 | 0.78 | 0.71 | 0.45 | 0.74 | 0.72 | 0.48 |
| Telfair | 1.43 | 1.11 | 0.73 | 0.75 | 0.56 | 0.80 | 0.74 | 0.47 | 0.64 | 0.65 | 0.34 |
| Terrell | 1.57 | 1.04 | 0.70 | 0.62 | 0.46 | 0.70 | 0.70 | 0.30 | 0.83 | 0.56 | 0.29 |
| Thomas | 1.41 | 0.99 | 0.62 | 0.56 | 0.44 | 0.69 | 0.59 | 0.34 | 0.61 | 0.59 | 0.35 |
| Tift | 1.85 | 1.13 | 0.71 | 0.72 | 0.46 | 0.79 | 0.61 | 0.37 | 0.70 | 0.67 | 0.39 |
| Toombs | 1.72 | 1.07 | 0.68 | 0.63 | 0.49 | 0.66 | 0.52 | 0.45 | 0.72 | 0.59 | 0.32 |
| Towns | 1.09 | 0.82 | 0.51 | 0.43 | 0.29 | 0.45 | 0.37 | 0.21 | 0.56 | 0.42 | 0.25 |
| Treutlen | 1.42 | 1.06 | 0.80 | 0.83 | 0.54 | 0.91 | 0.64 | 0.47 | 0.75 | 0.71 | 0.48 |
| Troup | 1.79 | 1.17 | 0.75 | 0.66 | 0.43 | 0.80 | 0.62 | 0.44 | 0.75 | 0.70 | 0.39 |
| Turner | 1.78 | 1.08 | 0.78 | 0.74 | 0.62 | 0.70 | 0.63 | 0.36 | 0.77 | 0.77 | 0.41 |
| Twiggs | 1.65 | 1.12 | 0.76 | 0.72 | 0.51 | 0.81 | 0.66 | 0.52 | 0.76 | 0.66 | 0.38 |
| Union | 1.20 | 0.85 | 0.55 | 0.49 | 0.30 | 0.54 | 0.43 | 0.25 | 0.53 | 0.47 | 0.24 |
| Upson | 1.74 | 1.16 | 0.67 | 0.64 | 0.50 | 0.73 | 0.67 | 0.51 | 0.73 | 0.65 | 0.42 |
| Walker | 1.45 | 1.08 | 0.70 | 0.70 | 0.41 | 0.76 | 0.59 | 0.42 | 0.69 | 0.66 | 0.39 |
| Walton | 2.09 | 1.38 | 0.88 | 0.70 | 0.48 | 0.84 | 0.72 | 0.53 | 0.84 | 0.66 | 0.44 |
| Ware | 1.43 | 1.01 | 0.74 | 0.66 | 0.47 | 0.73 | 0.61 | 0.38 | 0.73 | 0.62 | 0.33 |
| Warren | 1.55 | 1.13 | 0.65 | 0.72 | 0.45 | 0.65 | 0.64 | 0.35 | 0.71 | 0.63 | 0.51 |
| Washington | 1.52 | 1.17 | 0.79 | 0.58 | 0.36 | 0.78 | 0.64 | 0.36 | 0.80 | 0.67 | 0.34 |
| Wayne | 1.61 | 1.06 | 0.77 | 0.59 | 0.53 | 0.72 | 0.67 | 0.51 | 0.70 | 0.72 | 0.47 |
| Webster | 1.26 | 0.99 | 0.82 | 0.55 | 0.55 | 0.57 | 0.82 | 0.29 | 0.75 | 0.57 | 0.21 |
| Wheeler | 1.40 | 1.04 | 0.80 | 0.78 | 0.47 | 0.84 | 0.66 | 0.39 | 0.77 | 0.59 | 0.38 |
| White | 1.86 | 1.20 | 0.76 | 0.68 | 0.45 | 0.83 | 0.63 | 0.50 | 0.77 | 0.64 | 0.43 |
| Whitfield | 1.84 | 1.27 | 0.83 | 0.78 | 0.48 | 0.80 | 0.64 | 0.44 | 0.82 | 0.69 | 0.47 |
| Wilcox | 1.36 | 1.19 | 0.69 | 0.55 | 0.44 | 0.72 | 0.62 | 0.41 | 0.73 | 0.72 | 0.37 |
| Wilkes | 1.48 | 0.96 | 0.60 | 0.51 | 0.67 | 0.65 | 0.46 | 0.22 | 0.63 | 0.55 | 0.19 |
| Wilkinson | 1.73 | 1.13 | 0.85 | 0.59 | 0.55 | 0.79 | 0.62 | 0.49 | 0.91 | 0.69 | 0.42 |
| Worth | 1.64 | 0.91 | 0.74 | 0.61 | 0.49 | 0.73 | 0.60 | 0.43 | 0.69 | 0.59 | 0.37 |

Peak Infection Percentage in each county in the State of Georgia under all scenarios tested with urban counties highlighted in green.

**References**

1. Keskinocak P, Oruc BE, Baxter A, Asplund J, Serban N. American Community Survey,

2017 American Community Survey 5-year Estimates. U.S. Census Bureau2017.

2. Keskinocak P, Oruc BE, Baxter A, Asplund J, Serban N. Coronavirus (Covid-19) Data in the United States. The New York Times; 2020.

3. Keskinocak P, Oruc BE, Baxter A, Asplund J, Serban N. Census Summary File 1. U.S. Census Bureau; 2010.

4. Keskinocak P, Oruc BE, Baxter A, Asplund J, Serban N. Census Transportation

Planning Products, 5-year data. U.S. Census Bureau; 2016.

5. Health GDoP. Georgia Department of Public Health COVID-19 Daily Status Report 2020 [updated 14 April 2020; cited 2020 14 April 2020]. Available from: <https://dph.georgia.gov/covid-19-daily-status-report>.

6. Wu JT, Riley S, Fraser C, Leung GM. Reducing the Impact of the Next Influenza Pandemic Using Household-Based Public Health Interventions. PLOS Medicine. 2006;3(9):e361. doi: 10.1371/journal.pmed.0030361.

7. Shi P, Keskinocak P, Swann JL, Lee BY. The impact of mass gatherings and holiday traveling on the course of an influenza pandemic: a computational model. BMC Public Health. 2010;10(1):778. Epub 2010/12/24. doi: 10.1186/1471-2458-10-778. PubMed PMID: 21176155; PubMed Central PMCID: PMCPMC3022852.

8. Ekici A, Keskinocak P, Swann JL. Modeling Influenza Pandemic and Planning Food Distribution. Manufacturing & Service Operations Management. 2014;16(1):11-27. doi: 10.1287/msom.2013.0460.

9. Li Z, Swann JL, Keskinocak P. Value of inventory information in allocating a limited supply of influenza vaccine during a pandemic. PLoS One. 2018;13(10):e0206293. Epub 2018/10/26. doi: 10.1371/journal.pone.0206293. PubMed PMID: 30359445; PubMed Central PMCID: PMCPMC6201932.

10. Mizumoto K, Kagaya K, Zarebski A, Chowell G. Estimating the asymptomatic proportion of coronavirus disease 2019 (COVID-19) cases on board the Diamond Princess cruise ship, Yokohama, Japan, 2020. Eurosurveillance. 2020;25(10):2000180. doi: doi:<https://doi.org/10.2807/1560-7917.ES.2020.25.10.2000180>.

11. Mandavilli A. Infected but Feeling Fine: The Unwitting Coronavirus Spreaders: The New York Times; 2020 [31 March 2020].

12. Andrei M. Iceland’s testing suggests 50% of COVID-19 cases are asymptomatic ZME Science2020 [updated 26 March 202014 April 2020]. Available from: <https://www.zmescience.com/medicine/iceland-testing-covid-19-0523/>.

13. Day M. Covid-19: four fifths of cases are asymptomatic, China figures indicate. BMJ. 2020;369:m1375. doi: 10.1136/bmj.m1375.

14. Nishiura H, Kobayashi T, Suzuki A, Jung SM, Hayashi K, Kinoshita R, et al. Estimation of the asymptomatic ratio of novel coronavirus infections (COVID-19). International Journal of Infectious Diseases. 2020. Epub 2020/03/18. doi: 10.1016/j.ijid.2020.03.020. PubMed PMID: 32179137.

15. Team CC-R. Severe Outcomes Among Patients with Coronavirus Disease 2019 (COVID-19) — United States, February 12–March 16, 2020. Morbidity and Mortality Weekly Report (MMWR): CDC, 18 March 2020. Report No.

16. Walker PG, Whittaker C, Watson O, Baguelin M, Ainslie KEC, Bhatia S, et al. The Global Impact of COVID-19 and Strategies for Mitigation and Suppression. Imperial College COVID-19 Response Team, 2020.

17. Li R, Pei S, Chen B, Song Y, Zhang T, Yang W, et al. Substantial undocumented infection facilitates the rapid dissemination of novel coronavirus (SARS-CoV2). Science. 2020:eabb3221. Epub 2020/03/18. doi: 10.1126/science.abb3221. PubMed PMID: 32179701.

18. WHO. Report of the WHO-China Joint Mission on Coronavirus Disease 2019 (COVID-19) World Health Organization, 2020 16-24 February 2020. Report No.

19. Chen TM, Rui J, Wang QP, Zhao ZY, Cui JA, Yin L. A mathematical model for simulating the phase-based transmissibility of a novel coronavirus. Infectious Diseases of Poverty. 2020;9(1):24. Epub 2020/03/01. doi: 10.1186/s40249-020-00640-3. PubMed PMID: 32111262; PubMed Central PMCID: PMCPMC7047374.

20. Linton NM, Kobayashi T, Yang Y, Hayashi K, Akhmetzhanov AR, Jung S-m, et al. Incubation Period and Other Epidemiological Characteristics of 2019 Novel Coronavirus Infections with Right Truncation: A Statistical Analysis of Publicly Available Case Data. medRxiv. 2020:2020.01.26.20018754. doi: 10.1101/2020.01.26.20018754.

21. Ferguson NM, Laydon D, Nedjati-Gilani G, Imai N, Ainslie K, Baguelin M, et al. Impact of non-pharmaceutical interventions (NPIs) to reduce COVID19 mortality and healthcare demand. Imperial College London, Team ICC-R; 2020 16 March 2020. Report No.

22. Weitz J. Intervention Serology and Interaction Substitution: Exploring the Role of `Immune Shielding' in Reducing COVID-19 Epidemic Spread 2020.

23. Riou J, Hauser A, Counotte MJ, Althaus CL. Adjusted age-specific case fatality ratio during the COVID-19 epidemic in Hubei, China, January and February 2020. medRxiv. 2020:2020.03.04.20031104. doi: 10.1101/2020.03.04.20031104.

24. Ganyani T, Kremer C, Chen D, Torneri A, Faes C, Wallinga J, et al. Estimating the generation interval for COVID-19 based on symptom onset data. medRxiv. 2020:2020.03.05.20031815. doi: 10.1101/2020.03.05.20031815.

25. Zhou F, Yu T, Du R, Fan G, Liu Y, Liu Z, et al. Clinical course and risk factors for mortality of adult inpatients with COVID-19 in Wuhan, China: a retrospective cohort study. The Lancet. 2020;395(10229):1054-62. doi: 10.1016/s0140-6736(20)30566-3.

26. Xie J, Tong Z, Guan X, Du B, Qiu H, Slutsky AS. Critical care crisis and some recommendations during the COVID-19 epidemic in China. Intensive Care Medicine. 2020. doi: 10.1007/s00134-020-05979-7.

27. Georgia TSo. Executive Order to Ensure a Safe & Healthy Georgia: The State of Georgia; 2020 [cited 2020 24 April 2020]. Available from: <https://gov.georgia.gov/document/2020-executive-order/04022001/download>.

28. Development GDoE. Governor Kemp’s Statewide Executive Order: Guidelines for Businesses Georgia Department of Economic Development2020 [cited 2020 24 April 2020]. Available from: <https://www.georgia.org/covid19bizguide>.
