# Supplementary figures and images for: The impact of social distancing on COVID19 spread: State of Georgia case study
Source: PLoS One. 2020 Oct 12;15(10):e0239798. doi: 10.1371/journal.pone.0239798 (PMC7549801; doi:10.1371/journal.pone.0239798)

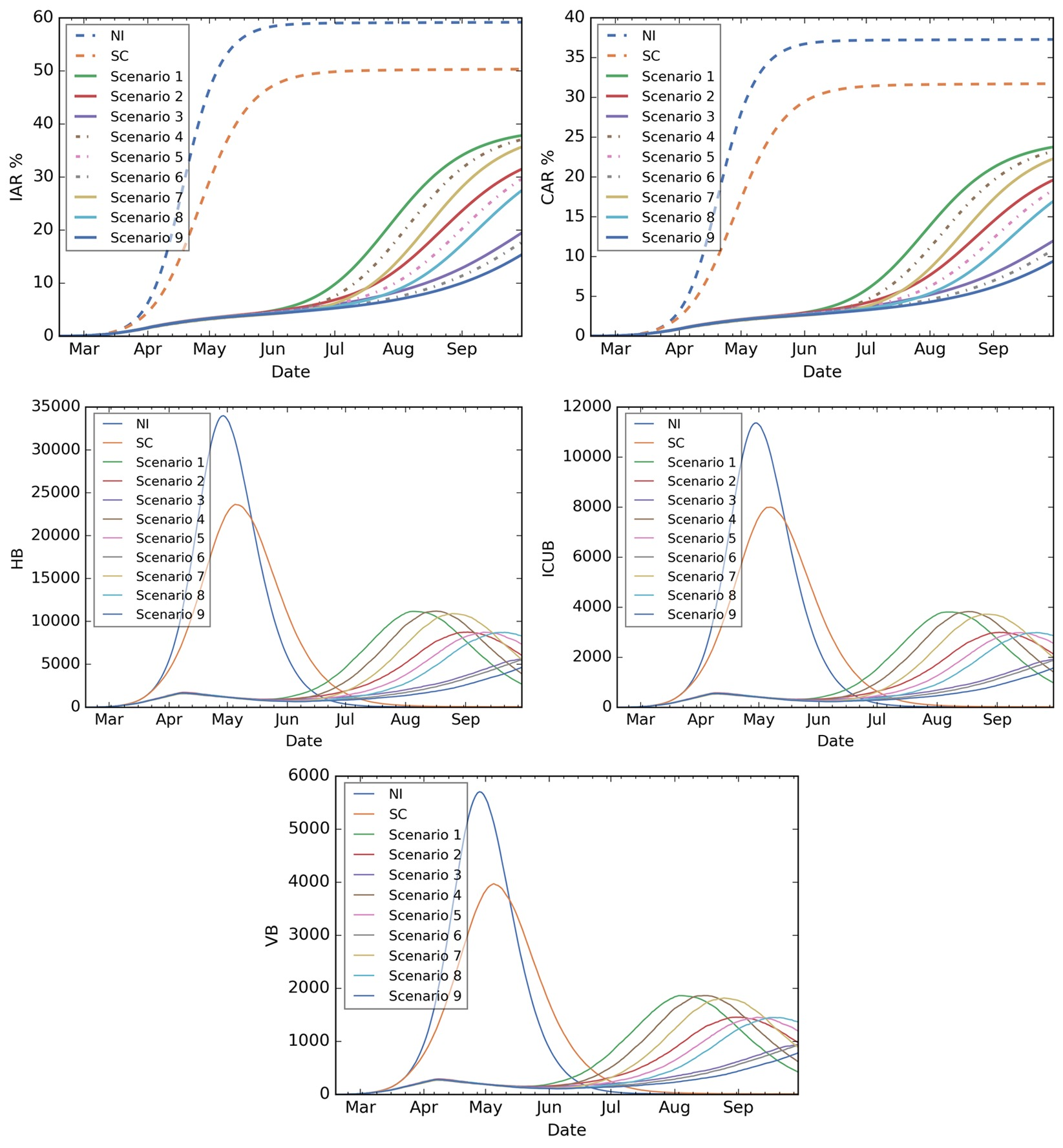

Supplement: S1 Fig — State Level Outcomes: IAR (first row left plot), CAR (first row right plot), HB (second row left plot), ICUB (second row right plot), V (third row center plot) across all scenarios (including the baseline scenarios). (TIF) [file pone.0239798.s002.tif]

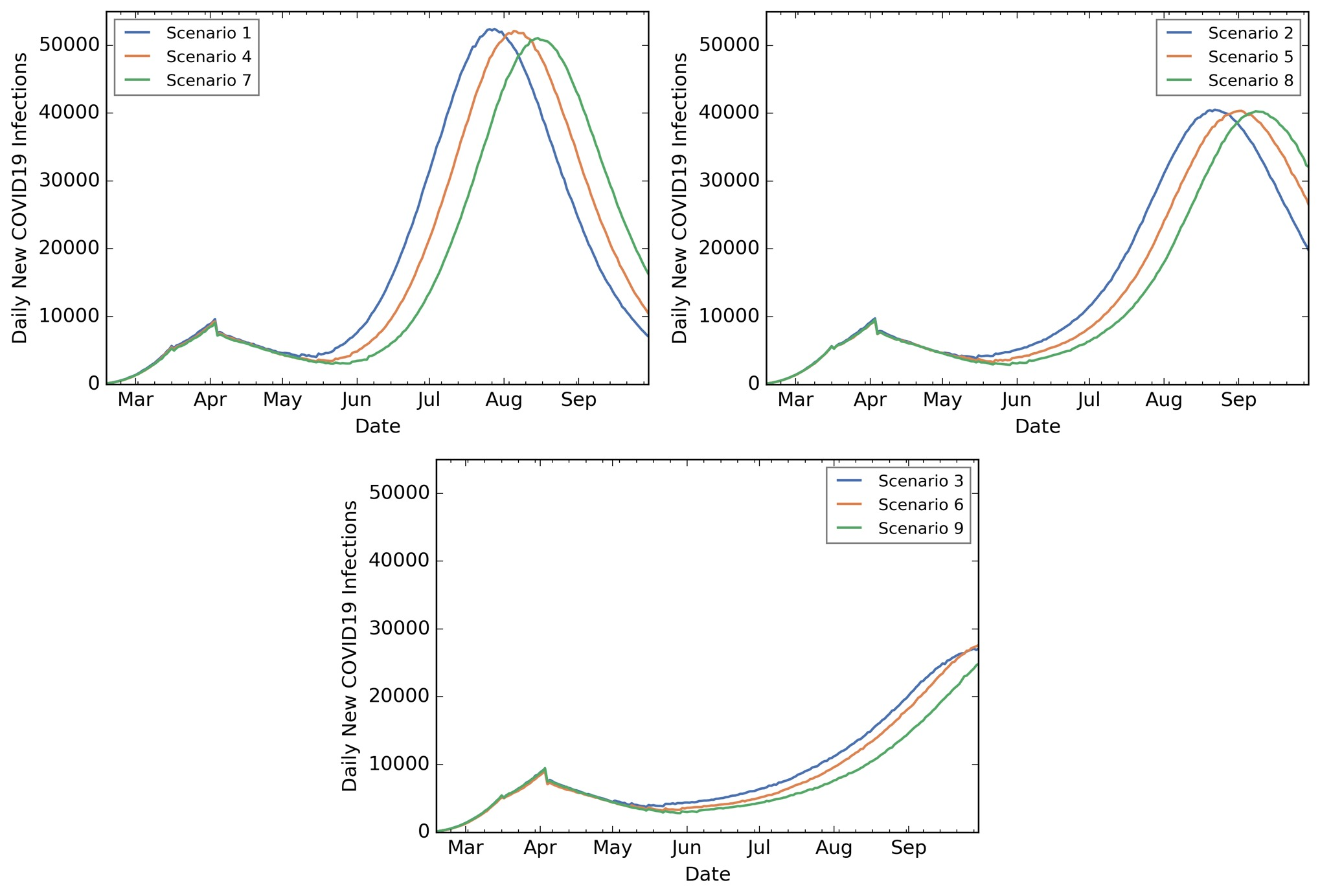

Supplement: S2 Fig — Daily new COVID19 infections when Low VQ is combined with 4 week (Scenario 1), 5 week (Scenario 4), 6 week (Scenario 7) SIP (top left plot), Medium VQ is combined with 4 week (Scenario 2), 5 week (Scenario 5), 6 week (Scenario 8) SIP (top right plot), High VQ is combined with 4 week (Scenario 3), 5 week (Scenario 6), 6 week (Scenario 9) SIP (bottom center plot). (TIF) [file pone.0239798.s003.tif]

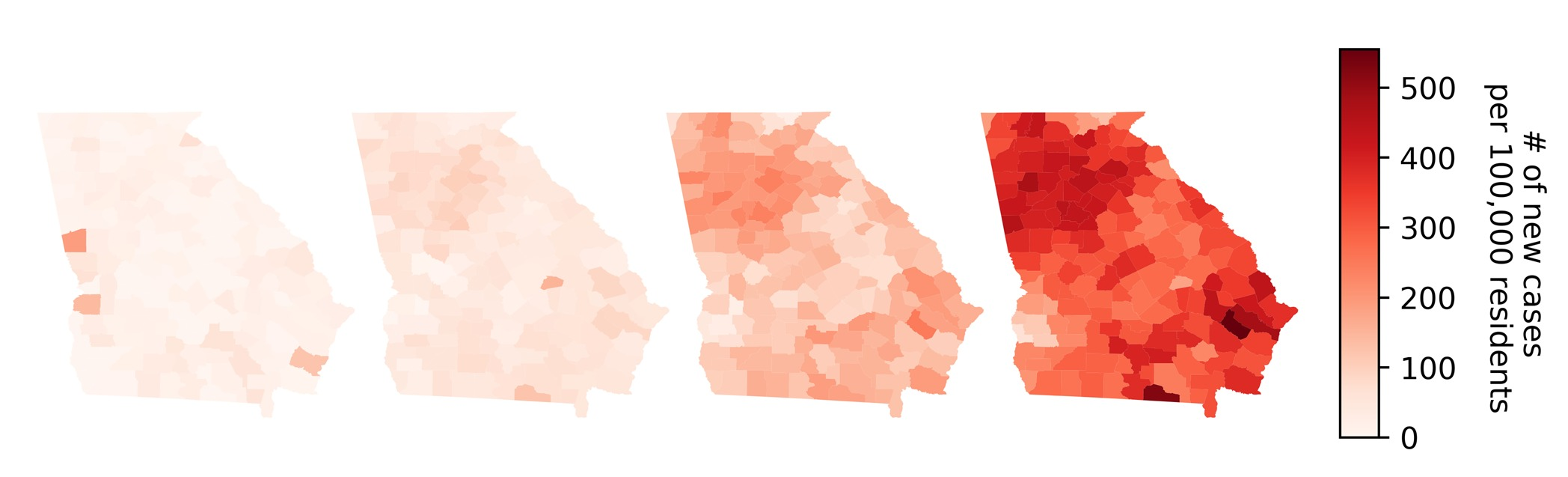

Supplement: S3 Fig — Four maps of Georgia at the county level recording the number of new infections per 100,000 people for June 23, 2020 (using the actual number of infections), July 15, 2020 (simulated data from our model), August 15, 2020 (simulated data), and September 15, 2020 (simulated data), respectively [59]. (TIF) [file pone.0239798.s004.tif]
